# SPP1^+^ TAM: CD8^+^ T Cell Crosstalk Associates with Blocking Radiotherapy Efficacy in Lung Cancer

**DOI:** 10.34133/research.0851

**Published:** 2025-08-25

**Authors:** Yuzhao Jin, Yuan Zhuang, Peng Luo, Xiaojie Zhang, Jinhua Luo, Yifei Ma, Dandan Guo, Na Hang, Qing Li, Zhijun Shen, Zhao Xie, Ruiqing Gao, Chenyu Gao, Ling Wang, Wenjun Mao, Bufu Tang, Songhua Cai

**Affiliations:** ^1^ Key Laboratory of Cancer Prevention and Intervention, Ministry of Education, The Second Affiliated Hospital, Zhejiang University School of Medicine; First Affiliated Hospital, Dalian Medical University, Dalian 116011, China.; ^2^ Department of Radiation Oncology, Zhongshan Hospital Affiliated to Fudan University, Shanghai, China.; ^3^ Department of Oncology, Zhujiang Hospital, Southern Medical University, Guangzhou, China.; ^4^ Wenzhou Medical University, Wenzhou 325000, China.; ^5^ Department of Pharmacy, China Medical University, Shenyang 110122, China.; ^6^ First Affiliated Hospital, Dalian Medical University, Dalian 116011, China.; ^7^ Department of Thoracic Surgery, The Affiliated Wuxi People’s Hospital of Nanjing Medical University, Wuxi People’s Hospital, Wuxi Medical Center, Nanjing Medical University, Wuxi, China.; ^8^ Department of Interventional Radiology, Zhongshan Hospital, Shanghai Institute of Medical Imaging, Shanghai Institution of Medical Imaging, Shanghai, National Clinical Research Center of Interventional Medicine, Fudan University, Shanghai 200032, China.; ^9^ National Cancer Center/National Clinical Research Center for Cancer/Cancer Hospital and Shenzhen Hospital, Chinese Academy of Medical Sciences and Peking Union Medical College, Shenzhen 518116, China.

## Abstract

Radiotherapy (RT) is a cornerstone treatment for non-small cell lung cancer (NSCLC), but its efficacy is often limited by immune suppression in the tumor microenvironment. In this study, we identified SPP1 as a key factor up-regulated after RT, mainly expressed by immunosuppressive macrophages. Single-cell RNA sequencing and in vivo models showed that SPP1^+^ macrophages inhibit CD8^+^ T cell infiltration and correlate with poor prognosis. Targeting SPP1 in macrophages enhanced RT efficacy, reduced tumor burden, and restored antitumor immunity. In summary, combining RT with SPP1^+^ macrophage-targeted intervention may serve as a promising strategy to overcome immune-mediated radioresistance and enhance therapeutic efficacy in NSCLC.

Radiotherapy (RT) is a cornerstone treatment for non-small cell lung cancer (NSCLC), yet tumor hypoxia and DNA repair mechanisms often contribute to resistance [1]. Recent studies suggest that RT can reshape the tumor microenvironment (TME) and modulate immune responses, potentially enhancing its efficacy when combined with immunomodulatory strategies [2,3]. Secreted phosphoprotein 1 (SPP1), expressed by tumor-associated macrophages (TAMs), contributes to tumor progression and immune suppression, and its high expression correlates with poor prognosis in cancers [4]. This study investigates how RT affects SPP1 expression in macrophages and explores the potential of targeting SPP1 to overcome RT resistance in lung cancer.

We first compared gene expression in lung tumors before and after RT, revealing an up-regulation of SPP1 following irradiation (Fig. 1A). Immune cell infiltration analysis indicated an increase in macrophage abundance after RT, with SPP1 expression positively correlating with macrophage infiltration (Fig. 1B and Fig. S1A to D). Immunofluorescence staining in a mouse model confirmed elevated levels of SPP1 and CD68 after RT (Fig. 1C). Functional enrichment analysis showed activation of extracellular matrix–receptor interaction and adhesion pathways following RT, linking SPP1 to RT resistance (Fig. S1E and F).

**Fig. 1.**
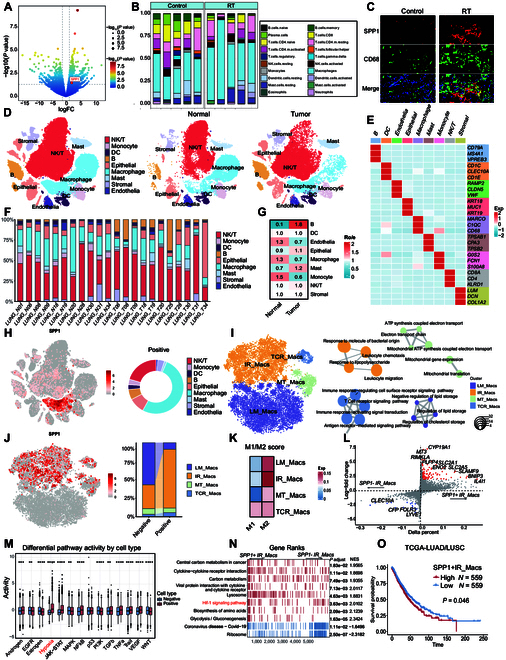
SPP1 up-regulation in macrophages following RT and its correlation with immune-suppressive function. (A) Gene expression comparison of lung cancer tumors before and after RT, with significant up-regulation of SPP1 after RT. (B) CIBERSORT analysis revealing increased macrophage abundance post-RT. (C) Immunofluorescence co-staining of SPP1 and CD68 up-regulation after RT. (D) Single-cell RNA sequencing identifying 9 distinct immune cell subpopulations in the lung cancer microenvironment. (E) Marker gene expression in distinct cell subpopulations. (F) Distribution of cell subpopulations across individual lung cancer samples. (G) Distribution of cell subpopulations across different tissue types. (H) Expression of SPP1 in various cell types. (I) Macrophage subpopulations based on functional gene expression patterns. (J) Expression of SPP1 in different macrophage subpopulations. (K) M1/M2 polarization scores in macrophage subpopulations. (L) Differentially expressed genes in SPP1^+^IR_Macs and SPP1^−^IR_Macs. (M) Pathway activity analysis showing elevated activity in key signaling pathways in SPP1^+^ IR_Macs. (N) GSEA enrichment of differentially expressed genes between SPP1^+^IR_Macs and SPP1^−^IR_Macs. (O) Prognostic analysis of SPP1^+^IR_Macs in TCGA-LUAD and TCGA-LUSC cohorts.

Single-cell RNA sequencing (RNA-seq) of lung cancer identified 9 distinct subpopulations, including macrophages, with varying distributions (Fig. 1D to G). SPP1 was highly expressed in macrophages, especially in tumor tissues (Fig. 1H). Macrophages were further categorized into 4 subpopulations: immune-related macrophages (IR_Macs), TCR-positive macrophages (TCR_Macs), lipid metabolism-associated macrophages (LM_Macs), and mitochondria-related macrophages (MT_Macs) (Fig. 1I). Notably, SPP1 was predominantly expressed in IR_Macs, which exhibited the highest M2 (anti-inflammatory) score, suggesting SPP1’s role in macrophage-mediated immunosuppression (Fig. 1J and K). Differential gene expression analysis showed that IL4I1 was more highly expressed in SPP1^+^ IR macrophages, correlating with T cell immunosuppression (Fig. 1L). Enrichment analysis of the differentially expressed gene set also revealed that SPP1^+^ IR macrophages were more active in several key pathways, including hypoxia, JAK-STAT, MAPK, PI3K, and TGF-β (Fig. 1M and N). Hypoxic tumor regions, enriched in SPP1^+^ macrophages, foster an immunosuppressive and tissue-repairing microenvironment, which impairs RT efficacy [5]. Additionally, the abundance of SPP1^+^ IR macrophages correlated with worse prognosis in The Cancer Genome Atlas (TCGA) cohorts (Fig. 1O).

T cells are essential effectors of antitumor immunity. Therefore, we investigated the relationship between SPP1^+^ IR macrophages and T cell subsets. We performed dimensionality reduction and clustering on NK/T cells, identifying 7 subpopulations: XCL1^+^ NK cells, FCGR3A^+^ NK cells, IL7R^+^ CD4^+^ naive T cells, CTLA4^+^ regulatory T cells (Tregs), ZNF683^+^ CD8^+^ memory T cells, ISG^+^ CD8^+^ T cells, and GZMK^+^ CD8^+^ effector T cells (Fig. S2A to D). Cell-type correlation analysis revealed a negative correlation between SPP1^+^ IR macrophages and T cell subsets, suggesting an immunosuppressive role (Fig. S2E). Communication analysis demonstrated strong interactions between SPP1^+^ IR macrophages and T cells, particularly in tumor tissues, with the SPP1–CD44 axis showing the strongest interaction (Fig. S3A and B). SPP1^+^ IR macrophages interacted with T cells via the macrophage migration inhibitory factor (MIF)–CD74–CD44/CXCR4 axis (Fig. 2A). The MIF signaling pathway, which is linked to poor prognosis and immune evasion, exhibited the second-highest interaction strength after the MHC pathway [6] (Fig. S3C). This signal was predominantly secreted by SPP1^+^ IR macrophages, with GZMK^+^ CD8^+^ T cells as the primary recipients (Fig. 2B).

**Fig. 2.**
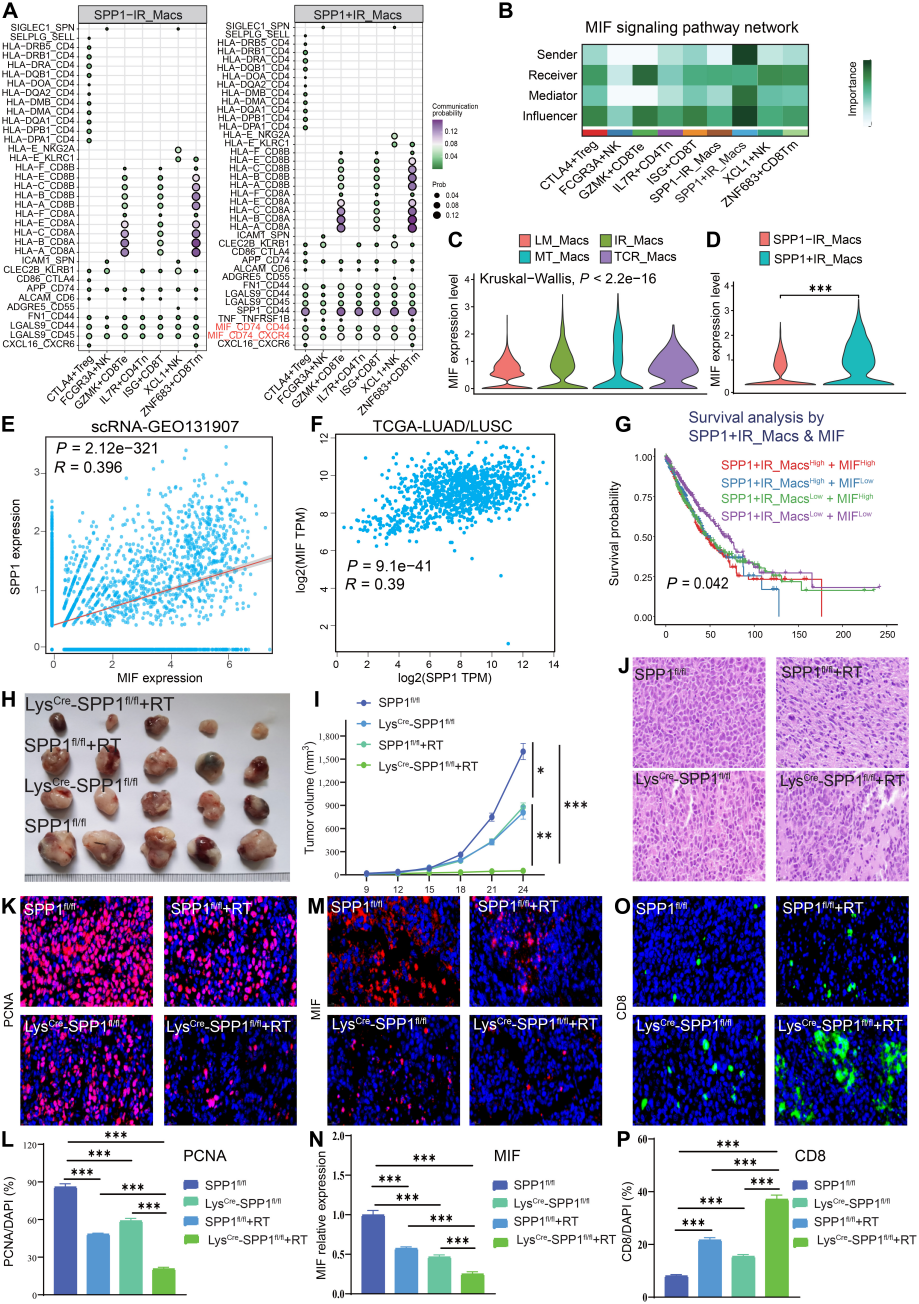
Targeting SPP1^+^ TAMs enhances antitumor immunity when combined with RT. (A) Dot plots showing communication probabilities between SPP1^+^ IR_Macs and other immune cells. (B) MIF signaling pathway network highlighting the role of SPP1^+^IR_Macs. (C) Violin plot comparing MIF expression levels in macrophage subpopulations. (D) MIF expression comparison between SPP1^+^ and SPP1^−^IR_Macs. (E) Correlation between SPP1 and MIF expression in GEO0131907. (F) Correlation between SPP1 and MIF expression in TCGA-LUAD/LUSC. (G) Survival analysis of SPP1^+^IR_Macs and MIF in TCGA-LUAD and TCGA-LUSC cohorts. (H) Tumor from different mouse groups. (I) Tumor volume growth curves of the different groups. (J) HE staining of tumor morphology. (K) PCNA immunofluorescence staining in tumors. (L) Quantification of PCNA-positive cells. (M) MIF immunofluorescence staining in tumors. (N) Quantification of MIF expression. (O) CD8^+^ T cell immunofluorescence staining in tumors. (P) Quantification of CD8^+^ T cells.

MIF expression was significantly higher in SPP1^+^ IR macrophages compared to SPP1^−^ IR macrophages (Fig. 2C and D). A strong positive correlation between SPP1 and MIF expression was observed at the single-cell level (Fig. 2E) and in bulk RNA-seq data from the TCGA cohorts (Fig. 2F). Patients with high expression of both SPP1^+^ IR macrophage signature and MIF showed significantly worse overall survival, highlighting their cooperative role in promoting an immunosuppressive microenvironment (Fig. 2G).

Taken together, our findings suggest that combining RT with strategies targeting SPP1^+^ macrophage-mediated immunosuppression may enhance CD8^+^ T cell-mediated antitumor immunity. To validate the potential of combining RT with targeting SPP1^+^ macrophages as a therapeutic strategy, we first generated the Lys^Cre^-SPP1^fl/fl^ mouse model. The design strategy is illustrated in Fig. S4A, and the knockout efficiency is shown in Fig. S4B. The mice were divided into 4 groups: SPP1^fl/fl^, Lys^Cre^-SPP1^fl/fl^, SPP1^fl/fl^ + RT, and Lys^Cre^-SPP1^fl/fl^ + RT. The experimental workflow is shown in Fig. S4C. The results showed that both SPP1 knockout in macrophages and RT alone inhibited tumor growth, while the combination treatment exhibited a significantly stronger antitumor effect (Fig. 2H and I). Hematoxylin–eosin (HE) staining and PCNA immunohistochemistry further supported the enhanced antitumor efficacy of the combination therapy (Fig. 2J to L). We also examined the relationship among SPP1^+^ macrophages, MIF, and CD8^+^ T cells. Knockout of SPP1 in macrophages significantly reduced MIF expression and increased CD8 expression. This effect became more pronounced after RT (Fig. 2M to P). Based on these observations, we speculate that targeting SPP1 can significantly suppress the expression of MIF, thereby reducing immunosuppressive signals and subsequently activating the function of CD8^+^ T cells. These findings suggest that targeting SPP1^+^ macrophages combined with RT can enhance antitumor immune responses.

Our results indicate that targeting SPP1^+^ macrophages represents a promising radiosensitizing strategy in NSCLC by reducing macrophage-mediated immunosuppression and enhancing CD8^+^ T cell infiltration, thereby improving RT efficacy. Combining RT with SPP1-targeted interventions could overcome radioresistance and potentially improve clinical outcomes. Although our results provide valuable mechanistic insights, further exploration is needed for clinical translation. Nanoparticle systems, such as CANDI460, have shown success in targeting SPP1 to inhibit tumor growth [7]. Macrophage targeting specificity is also crucial, and antibody–drug conjugates or engineered nanoparticles targeting TAMs may enhance specificity [8,9].

While our study provides valuable insights using mouse models and public datasets, these models may not fully replicate the complexity of the human TME. Future research should incorporate more clinically relevant models, such as patient-derived organoids or humanized mice, to better evaluate the efficacy and toxicity of SPP1-targeted therapies. Additionally, optimizing SPP1 inhibitors and delivery systems, such as nanoparticle-based platforms, and exploring combinations with immune checkpoint inhibitors or chemotherapy could enhance RT efficacy. Addressing other immune-suppressive mechanisms, like myeloid-derived suppressor cells (MDSCs), Tregs, or mregDC, may also improve therapeutic outcomes [10]. In conclusion, targeting SPP1^+^ macrophages shows promise for enhancing RT efficacy in NSCLC and overcoming radioresistance.

## Ethical Approval

All animal experiments conducted in this study were approved by the Ethics Committee of the Cancer Hospital Chinese Academy of Medical Sciences (NCC2024A629).

## Data Availability

The sequencing data of NSCLC before and after radiotherapy were obtained from GSE162945. Single-cell RNA sequencing of lung cancer was obtained from GSE131907. RNA expression and clinical survival data of the TCGA-LUAD and TCGA-LUSC cohorts were obtained from the GDC database (https://gdc.cancer.gov/).
