# Supplementary material for: SPP1+ TAM: CD8+ T Cell Crosstalk Associates with Blocking Radiotherapy Efficacy in Lung Cancer
Source: Research (Wash D C). 2025 Aug 25;8:0851. doi: 10.34133/research.0851 (PMC12599977; doi:10.34133/research.0851)
Supplement: Supplementary 1 — Supplementary Text Figs. S1 to S4 [file research.0851.f1.doc]

**SPP1+TAM:CD8+ T cell crosstalk associates with blocking radiotherapy efficacy in lung cancer**

**Supplementary Materials**

**1.Supplementary figure legends**

**Figure S1 RT enhances macrophage infiltration and correlates with increased SPP1 expression** (A) Box plots comparing the proportion of immune cells in tumor tissues using CIBERSORT between control and RT-treated groups. (B) Correlation between SPP1 expression and macrophage proportion, showing a positive correlation (R = 0.6, p = 0.008). (C) Box plots comparing immune cell fractions in tumor tissues using ssGSEA. (D) Correlation between SPP1 expression and macrophage fraction from ssGSEA analysis, showing a positive correlation (R = 0.55, p = 0.019). (E) GO enrichment analysis of differential genes in lung cancer patients before and after RT. (F) KEGG enrichment analysis of differential genes in lung cancer patients before and after RT.

**Figure S2 T cell subset identification** (A) Dot plot showing the expression of marker genes across different T cell subsets. (B) GO enrichment analysis for different T cell subsets, highlighting pathways related to immune activation and regulation. (C) tSNE plot showing the clustering of T cell subsets. (D) The proportion of different T cell subsets in normal and tumor tissues. (E) Heatmap showing the correlation between different immune cell types and T cell subsets.

**Figure S3 Cell communication between T cells and macrophages** (A) Network diagram showing the number of interaction and interaction strength between T cell subsets and macrophages. (B) Dot plot illustrating the communication probability of various ligand-receptor pairs between T cell subsets and macrophages in normal and tumor tissues. (C) Heatmap showing the outgoing and incoming signaling patterns between T cell subsets and macrophages.

**Figure S4 SPP1 knockout validation and experimental workflow** (A) Schematic illustrating the generation of Lys^Cre^-SPP1^fl/fl^ mice by crossing Lys^Cre^ mice with SPP1^fl/fl^ mice. (B) PCR validation showing the presence of Lys Cre (left) and SPP1 loxP (right) alleles in the knockout mice. (C) Experimental timeline for the LLC cell injection, RT treatment (8Gy x 3), and tumor resection at day 24 in SPP1^fl/fl^ and Lys^Cre^-SPP1^fl/fl^ mice.

**2.Supplementary figures**

**Figure S1**

**
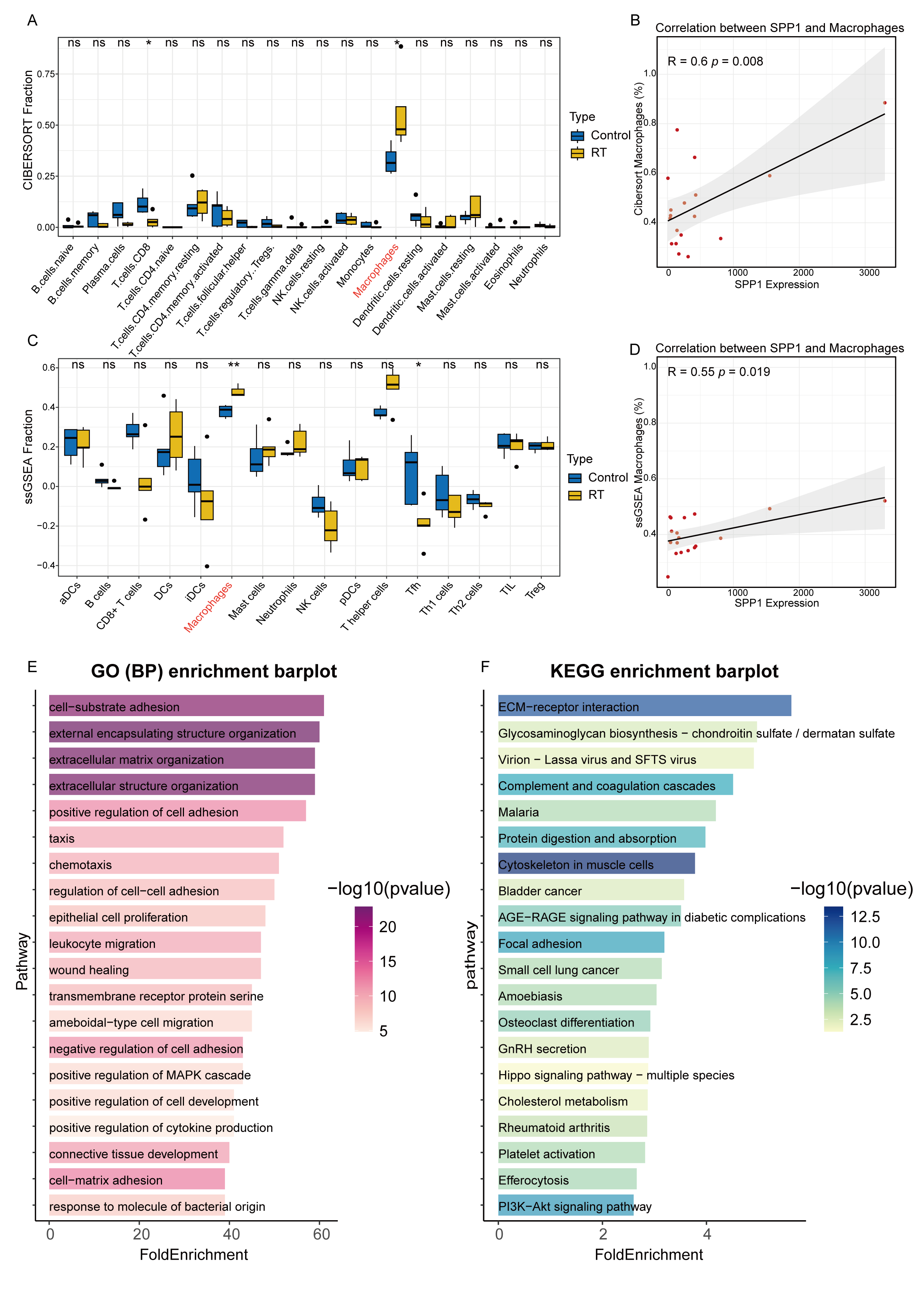
**

**Figure S2**

**
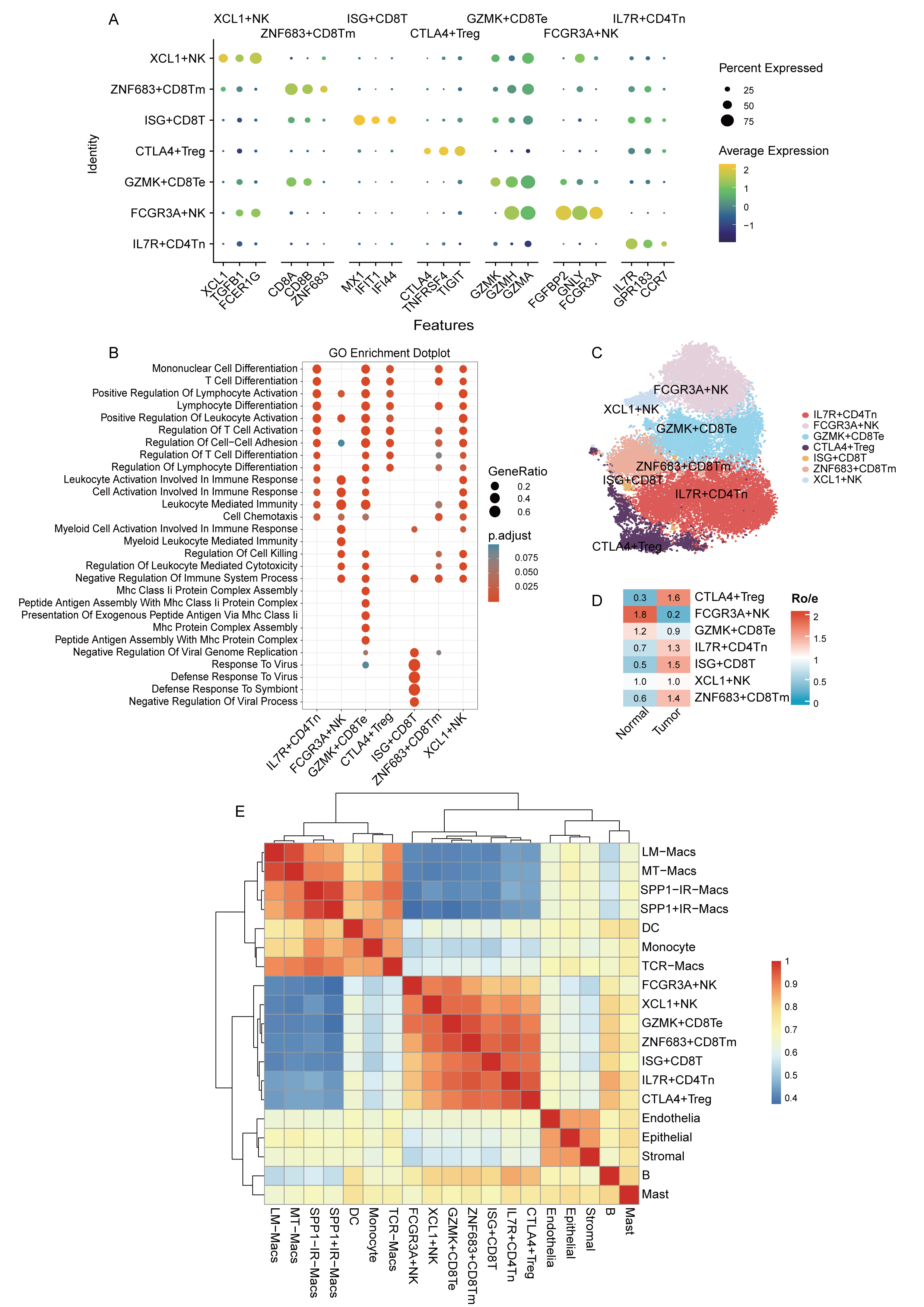
**

**Figure S3**

**
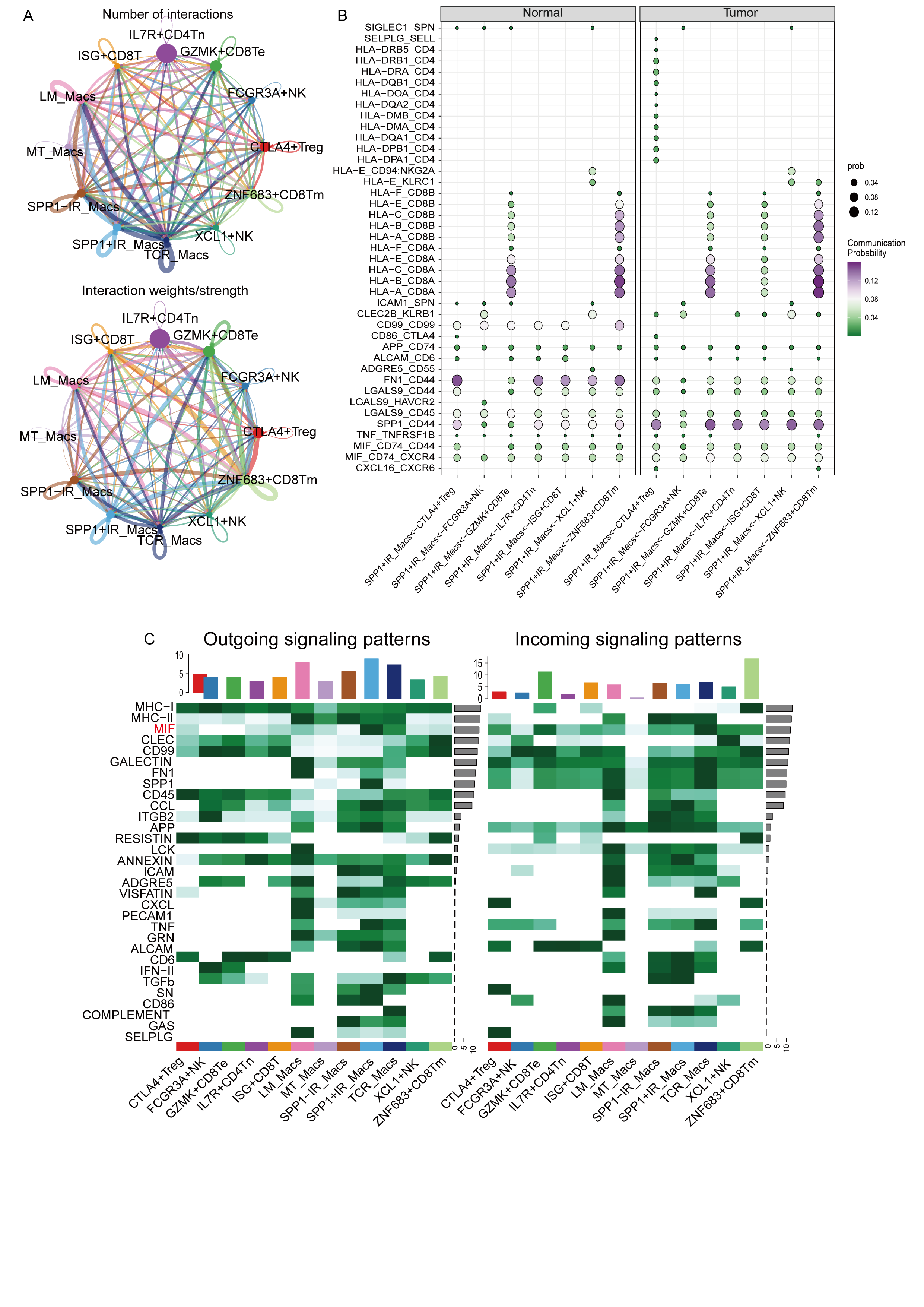
**

**Figure S4**

**
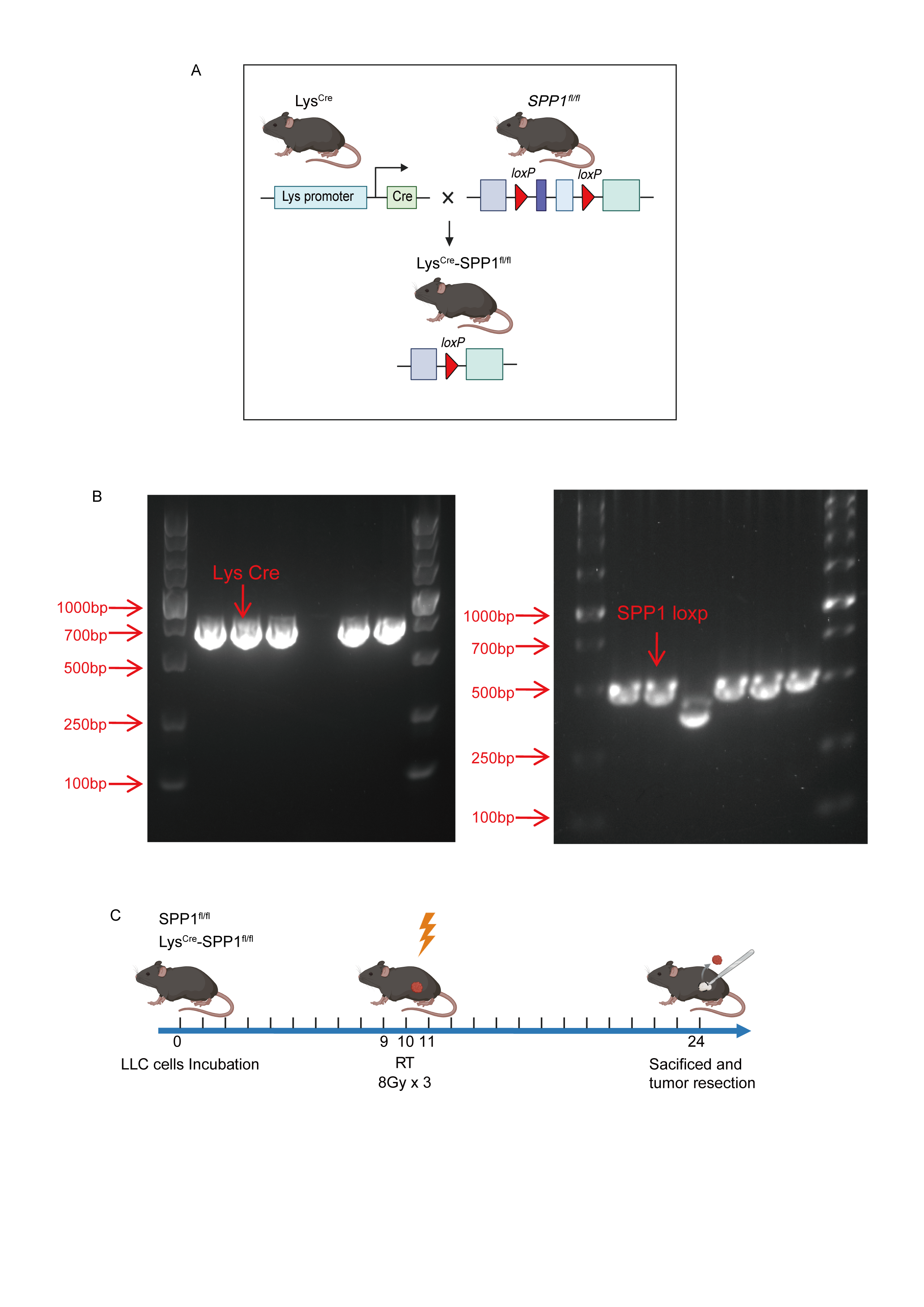
**

**3.Materials and Methods**

**Data acquisition**

The sequencing data of non-small cell lung cancer before and after radiotherapy was obtained from GSE162945, with metastatic samples removed and 10 primary samples retained. Single-cell RNA sequencing of lung cancer was obtained from GSE131907, with 11 tumor and 11 normal lung tissue samples retained. RNA expression and clinical survival data of the TCGA-LUAD and TCGA-LUSC cohorts were obtained from the GDC database (<https://gdc.cancer.gov/>).

**CIBERSORT and ssGSEA**

Processed GSE162945 matrix was analyzed with CIBERSORT using the LM22 gene signature, which includes 22 immune cell types, for cell type deconvolution. The results provide the relative abundance of each immune cell type in the samples.
For single-sample Gene Set Enrichment Analysis (ssGSEA), we evaluated the enrichment of predefined gene sets related to immune cells in each sample. The input consisted of normalized gene expression data. Enrichment scores for each gene set were calculated, representing the coordinated upregulation or downregulation of genes within the set in each sample. These scores were then used to compare the proportions of different cell types across the samples.

**Survival analysis**

We analyzed the overall survival of the TCGA-LUAD/LUSC cohorts based on the median value of the data, and Kaplan-Meier survival curves were generated using the survival package.

**scRNA data process**

The GSE131907 dataset was analyzed using the Seurat R package, with preprocessing steps designed to ensure high data quality. Cells were first filtered based on the following criteria: a minimum of 400 unique features expressed per cell, total RNA counts between 1,000 and 20,000, and mitochondrial gene content less than 10%. Seurat objects were created for both tumor and normal tissue samples. Data normalization was performed using the NormalizeData function, followed by scaling with the ScaleData function. The top 2,000 most variable genes were selected for downstream analysis, and principal component analysis was conducted. The optimal number of principal components was determined to be 30, based on elbow plot inspection.

To correct for batch effects, the Harmony algorithm was applied to adjust for technical variations across samples. Cell clustering was performed at a resolution of 1.0, and cell populations were manually annotated using known marker genes. The marker genes selected to define each cell population include CD79A, MS4A1, VPREB3 for B cells, CD1C, CD1E and CLEC10A for DC, RAMP2, CLDN5, and VWF for endothelial cells, KRT18, MUC1, and KRT19 for epithelial cells, MARCO, C1QC, and CD68 for macrophages, TPSAB1, CPA3, and TPSB2 for mast cells, G0S2, FCN1, and S100A8 for monocytes, CD8A, CD4, and KLRD1 for NK T cells (NKT), and LUM, DCN, and COL1A2 for stromal cells. Clusters were visualized using tSNE plots, and the intensity of red in the heatmap represents the expression levels of each marker gene within the respective cell populations. Higher expression is indicated by darker red, allowing for clear differentiation of the cell populations based on their unique marker profiles. Subsequent analysis focused on macrophages, NK / T cell subsets. These populations underwent a secondary normalization and processing pipeline. Clustering was then conducted at a finer resolution of 0.3, followed by manual annotation to assign specific cell population identities based on marker gene expression profiles.

All analyses were performed using the Seurat package in the R environment, with appropriate functions for data processing and visualization.

**Enrichment analysis**

GO enrichment analysis was performed using the enrichGO function from the clusterProfiler package to assess the enrichment of differentially expressed genes (DEGs) in biological processes. Gene identifiers were converted from gene symbols to ENTREZID using the org.Hs.eg.db database for annotation. The p-value and q-value cutoffs were set to 0.05 to filter significant pathways. Enrichment bar plots were generated using ggplot2, displaying the top 20 significantly enriched pathways based on the enrichment factor and p-values.

KEGG enrichment analysis was conducted using the enrichKEGG function to identify KEGG pathways associated with DEGs. The p-value and q-value cutoffs were set to 0.05 to select significant pathways. The fold enrichment for each pathway was calculated, and KEGG enrichment bar plots were created using ggplot2. The top 20 enriched pathways were selected and sorted based on their fold enrichment.

Gene set enrichment analysis (GSEA) was performed using the clusterProfiler package. Differentially expressed genes were first identified between the "Positive" and "Negative" groups based on the expression of SPP1. The log fold change threshold was set to 0.25, and only genes with an adjusted p-value < 0.05 were considered significant. These DEGs were ranked according to their log2 fold change values. The top DEGs were then used for GSEA analysis using the gseGO function to assess enrichment in biological processes using the GO database. The analysis was performed with a minimum gene set size of 10, a maximum gene set size of 500, and a p-value cutoff of 0.05. Enrichment results were visualized to identify significant biological processes associated with the DEGs.

**Cell communication analysis**

Utilize tools such as CellChat and CellPhoneDB to analyze cell-to-cell communication. Infer receptor-ligand interactions from GSE131907 data using established interaction databases. Extract expression data for receptor-ligand pairs and compute their interaction scores. Visualize the interaction network to illustrate communication patterns between different cell types or clusters.

**RT treatment**

Irradiation was performed using the SARRP3 X-ray biological irradiator (X trahl, USA) with a dose rate of 3.96 Gy/min. For the mouse irradiation experiments, a dose of 8 Gy was administered for three consecutive days.

**Mouse Model and Irradiation Procedure**

LLC lung cancer cells (1×10^6^/100 µL) were subcutaneously injected into the inner side of the left lower limb of 8-week-old male C57BL/6 mice. Once the tumor reached approximately 100 mm^3^, irradiation was performed using the SARRP3 X-ray biological irradiator (X trahl, USA) with a dose rate of 3.96 Gy/min. A single dose of 8 Gy was administered for three consecutive days.

Additionally,1×10^6^/100 µL LLC cells were subcutaneously injected into 8-week-old male Lys^Cre^-SPP1^fl/fl^ C57BL/6 mouse models and control SPP1^fl/fl^ C57BL/6 mice. The mice were divided into four groups: SPP1^fl/fl^, Lys^Cre^-SPP1^fl/fl^, SPP1^fl/fl^ + RT, and Lys^Cre^-SPP1^fl/fl^ + RT. On the ninth day, the same irradiation procedure was applied, with tumors measured the following day. Fluid volumes were monitored every three days thereafter. Mice were sacrificed on the 24nd day. After tumor removal, the tumors were photographed, weighed, fixed in formalin for 24 hours, dehydrated using a gradient ethanol series, embedded in paraffin, and sectioned for further staining.

Tumor volume was calculated using the formula:Tumor volume = (1/2×long diameter) × (short diameter)^2^. C57BL/6 male mice (6-8 weeks old) were obtained from the Shanghai Experimental Animal Center (Shanghai, China). Lys^Cre^-SPP1^fl/fl^ and control SPP1^fl/fl^ C57BL/6 mice were purchased from Cyagen Biosciences Inc. All mice were housed in accordance with SPF standards at 24℃±2℃, 40%-70% relative humidity, under a 12-hour light and 12-hour dark cycle, and were acclimatized before starting the experiments. All animal procedures were carried out according to protocols approved by the Fudan University Institutional Animal Care and Use Committee.

**Immunofluorescence Staining**

Tumor tissue blocks were sectioned into 4–6 µm thick slices and mounted on glass slides. The sections were dewaxed in xylene, followed by rehydration through a graded ethanol series. Antigen retrieval was performed using citrate buffer in a microwave oven. After cooling to room temperature, the sections were incubated with 5% BSA for 1 hour to block non-specific binding. Primary antibodies were applied and incubated overnight at 4℃. Following PBS washes, the sections were incubated with fluorescently labeled secondary antibodies for 1 hour at room temperature, shielded from light. Nuclei were stained with DAPI and the slides were mounted with antifade medium. Fluorescence images were obtained using a fluorescence microscope.

**Hematoxylin and Eosin (HE) Staining**

The tissue sections were deparaffinized in xylene and gradually rehydrated through a series of decreasing ethanol concentrations. They were then stained with hematoxylin for 5 minutes, washed in running tap water for 5 minutes, differentiated briefly in 1% acid alcohol, and blued with 0.2% ammonia water or saturated lithium carbonate solution. After rinsing in tap water, the sections were counterstained with eosin for 2 minutes. The stained sections were dehydrated through an ascending ethanol series, cleared in xylene, and mounted with a coverslip using synthetic resin. The slides were examined and captured using a light microscope.

**Statistical analysis**

Data were expressed as mean ± SEM. Kruskal.test was used to examine the MIF expression across multiple groups in the single-cell gene matrix, with the R packages ggpubr. The Wilcox test was used to examine MIF expression between two groups, using the R package ggpubr. ANOVA and Tukey's test were used to assess differences in data across multiple groups in the experiment. The two-tailed Student's t-test was used to examine data differences between two groups in the experiment. P values < 0.05 were considered significant (p<0.05*; p<0.01**, p<0.001***).

**4.Reagent Description**

**Antibodies**

| Name | Dilution | Species | Manufacture | Cat, No. |
| --- | --- | --- | --- | --- |
| CD68 | 1:100 | Rabbit | Abcam | ab283654 |
| Osteopontin | 1:50 | Rabbit | Proteintech | 22952-1-AP |
| PCNA | 1:400 | Rabbit | Cell Signaling  Technology | 13110 |
| CD8 | 1:100 | Rabbit | Abcam | ab217344 |
| MIF | 1:100 | Rabbit | Abcam | ab7207 |
